# ﻿Assessing ploidy levels and karyotype structure of the fire ant *Solenopsissaevissima* Smith, 1855 (Hymenoptera, Formicidae, Myrmicinae)

**DOI:** 10.3897/compcytogen.17.100945

**Published:** 2023-04-11

**Authors:** Ananda Ribeiro Macedo de Andrade, Danon Clemes Cardoso, Maykon Passos Cristiano

**Affiliations:** 1 Genetics and Evolution of Ants Research Group, Universidade Federal de Ouro Preto, Ouro Preto, MG – 35400-000, Brazil Universidade Federal de Ouro Preto Ouro Preto Brazil

**Keywords:** Evolution, Fire ant, Invasive species, Karyotype, Polyploidy

## Abstract

The family Formicidae is composed of ants that organize themselves into castes in which every individual has a joint organizational function. *Solenopsis* Westwood, 1840 is an ant genus with opportunistic and aggressive characteristics, known for being invasive species and stings that cause burning in humans. This genus is particularly difficult to classify and identify since its morphology provides few indications for species differentiation. For this, a tool that has been useful for evolutionary and taxonomic studies is cytogenetics. Here, we cytogenetically studied *Solenopsissaevissima* Smith, 1855 from Ouro Preto, Minas Gerais, Brazil. We evaluated the occurrence of polyploid cells in individuals and colonies by conventional cytogenetics. A total of 450 metaphases were analyzed and counted. Chromosome counts of individuals and colonies showed varied numbers of ploidies, from n = 16 to 8n = 128. The karyomorphometrical approach allowed determination of the following karyotypes: n = 10 m + 4 sm + 2 st, 2n = 20 m + 8 sm + 4 st, and 4n = 40 m + 16 sm + 8 st. Polyploidy can be found naturally in individuals and colonies and may represent an adaptative trait related to widespread distribution and invasion ability of new habitats.

## ﻿Introduction

Ants are recognized as some of the most successful organisms among invertebrates, being widely distributed throughout the world (Hölldobler and Wilson 1990). Formicidae is a clade that includes all ants and is the only family in which all species have the characteristic known as eusociality. This means that colonies are organized in castes, exhibit division of labor with overlapping generations (Wilson 1990). The queen, or sometimes queens in polygynous species, is responsible for reproduction, while the workers build the nest, defend the colony, and are responsible for obtaining and handling resources (Wilson 1998). The division of labor can also be related to the morphology of each worker, in which the sizes and ages of the workers will define which function they perform within the nest (Haight 2010).

Myrmicinae is the most diverse subfamily and includes the genus *Solenopsis* Westwood, 1840, which are known as “fire ants”. This popular name based on their aggressiveness and painful sting, which is due to the accumulation of allergenic proteins and alkaloids in their venom (Fox et al. 2010). They are native to South America (Buren 1972) but have great potential for habitat invasion. Some species of *Solenopsis* are currently found in Central America, North America, and Oceania (Callcott and Collins 1996; Holway et al. 2002).

Although ants are essential organisms within their ecosystems as they participate in maintaining the soil, nutrient cycling, and other ecosystem services (Lobry De Bruyn 1999), members of the genus *Solenopsis* are also responsible for great damage, both in agriculture and their effects on humans and animals, and can be considered pests (Santos 2016). The species *Solenopsissaevissima* Smith, 1855 is responsible for 35% of the reports of insect bites (Fox et al. 2012). They are also invasive, and when fire ants arrive in a new environment, they become harmful to other native species, which can be removed by competition due to the aggressiveness imposed by them (Wojcik et al. 2001).

The genus *Solenopsis* comprises more than 190 described species worldwide. They are cosmopolitan and taxonomically difficult. According to Fox et al. (2010), Pitts et al. (2005), and Shoemaker et al. (2006), workers lack morphological features for precise classification, and the morphological differences in some groups are not easily perceptible. In this context, cytogenetic and molecular data can provide useful markers for the systematics and taxonomy of this ant group.

Cytogenetics is a field of study interested in understanding the structure and function of the chromosomes (Speicher and Carter 2005). How the genome of an organism is organized into a defined number of DNA molecules is one of the most basic pieces of information that is reflected by the karyotype of the species. Thus, cytogenetic studies are relevant for evolutionary and taxonomic knowledge since the analysis of karyotypes can help distinguish species, and therefore complement phylogenetic and evolutionary analyses (Lukhtanov et al. 2006; Lorite and Palomeque 2010). For instance, a particular example of how cytogenetics can be used in the taxonomy of ants is the genus *Amoimyrmex* Cristiano, Cardoso et Sandoval, 2020 (Cristiano et al. 2020), i.e., a new genus of leaf-cutting ants discovered by integrating cytogenetics, molecular genetics, and morphology. Numerous other taxonomic issues for which cytogenetics could be useful are still to be addressed. Today, only 7% of ants have been cytogenetically analyzed (Cardoso et al. 2018a), which represents less than 1,000 species from more than 16,000 species known so far (Bolton 2022).

Even considering the small number of species cytogenetically studied, ants show an extreme karyotype diversity varying from the haploid number n = 1 (Crosland and Crozier 1986) to n = 60 chromosomes (Mariano et al. 2008). Considering only *Solenopsis*, two main karyotypes were recovered, n = 11 and n = 16 chromosomes (Cardoso et al. 2018a). Wurm et al. (2011) presented information about the genome of *Solenopsisinvicta* Buren, 1972, making it possible to understand its genomic structure, such as the identification of gene duplications and the multifunctionality of vitellogenin genes. Polyploid cells were already reported in insects and were suggested to be regulated by the endoreplication system (Fox and Duronio 2013). Endoreplication is a process that results in polytene chromosomes that have thousands of DNA strands. Polyploid organisms are common in plants (Otto and Whitton 2000), however they are rare in animals (White 1973; Clark and Wall 1996). Nevertheless, polyploidy is a heritable condition where an organism possess more than two complete sets of chromosomes. Polyploid cells can be identified through cytogenetic evidence and further confirmed by flow cytometry (FCM).

Polyploidy in ants has already been reported, but the studies do not describe whether and how the karyotype varies within the colony. In the present study, we describe the karyotype of the species *Solenopsissaevissima* from Brazil and evaluate whether and how the karyotype varies within individuals and the colony. We also perform a karyomorphometric analysis to precisely determine the karyotype structure and provide quantitative data for *S.saevissima* chromosomes. Additionally, we used flow cytometry analysis to determine the ploidy level of brain cells of *S.saevissima*. These data will certainly help our understanding of the ant’s genome evolution, taxonomy, and systematics.

## ﻿Material and methods

### ﻿Species sampling

**Figure 1.**
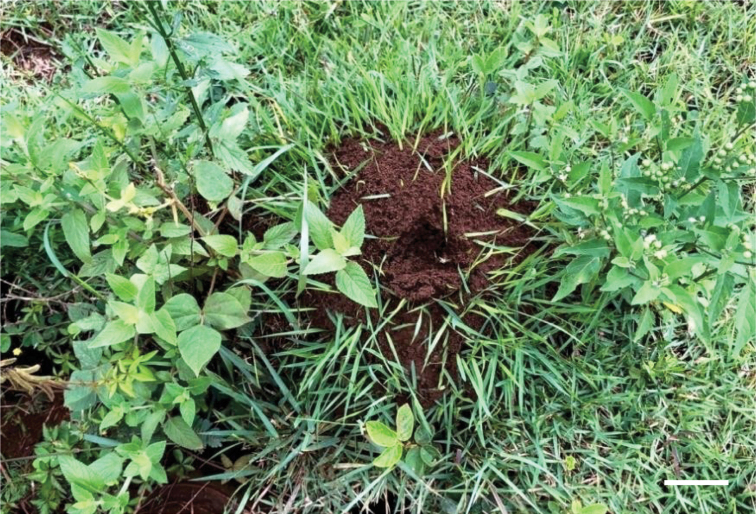
*Solenopsissaevissima* mound located in a grassy field in the campus of Morro do Cruzeiro, Universidade Federal de Ouro Preto, Ouro Preto - MG, Brazil. Scale bar: 3 cm.

*Solenopsissaevissima* colonies were sampled in Ouro Preto, Minas Gerais, Brazil (20°17'15"S, 43°30'29"W) located in the southeast region at over 1,150 m of altitude. Sampling occurred from October to December 2020, the period when broods were available. The nests were identified according to the description by Porter and Tschinkel (1987), who mentioned nests as mounds of soil located in grassy, sunny, open areas (Fig. 1). We marked the colonies as 1–4. The ants were collected with the aid of gloves and a shovel, stored in a plastic container, and taken to the laboratory for further processing. We never collected the entire colony, allowing the brood to recover.

### ﻿Sample preparation and obtention of mitotic cells

The colony fractions of *S.saevissima* were taken directly to the laboratory, and while alive, the post-defecating larvae (without meconium; or pre-pupae) were isolated. As described by Imai et al. (1988) and detailed by Cardoso et al. (2017), the cerebral ganglion of the larvae was removed and transferred to a container containing hypotonic colchicine solution (0.005% w/v colchicine in 1% sodium citrate solution) and incubated for 60 min in the dark. The time of incubation was adjusted considering the frequency of metaphases and standard condensation pattern (see Cristiano et al. 2017). Therefore, the ganglia were placed on a slide and smashed until smooth with the aid of two needles to release the cells. Metaphase spreads were obtained by dropping solutions on smashed tissue serially: first, solution 1 (acetic acid:ethanol:distilled water; 3:3:4), followed by solution 2 (acetic acid:ethanol; 1:1), and finally solution 3 (acetic acid 100%). After air drying, the slides were labeled with the respective colony code.

The slides were stained with Giemsa (4%) to observe the chromosomes under an optical microscope. Metaphases were photographed using a Zeiss Axio Imager Z2 microscope coupled to an AxioCam MRc image capture system. A total of 450 photos were captured of the metaphases found on the slides from the four different colonies (N1, N3, N4, and N5). The number of chromosomes was counted in all captured photos. A minimum of ten well-spread haploid (n) (males) and diploid metaphases (2n) (females) were assembled and submitted to karyomorphometrical analysis according to the description by Cristiano et al. (2017). For each chromosome, the total length (TL), short arm (S), and long arm (L) were measured, calculated as the distance between the arm telomere and the centromere. The total length (KL) of the karyotype was calculated from the sum of the total length (TL) of all chromosomes. The relative size (RL) was calculated in relation to the total size of all chromosomes with the formula (TL × 100 / ∑TL). The ratio (*r*) between the length of the long arm and short arm was given by the formula (*r* = L / S) and used to classify the chromosomes as metacentric (m), submetacentric (sm), and subtelocentric (st) as described by Levan et al. (1964).

Genome size (in picograms, pg) was estimated by flow cytometry in individuals from the four colonies following the protocol established by Moura et al. (2020). Cerebral ganglia of the post-defecating larvae from workers and the internal standard (*Drosophilamelanogaster*) were detached and immersed in 100–300 μL of Galbraith buffer and ground to release the cell nuclei. Subsequently, 600 μL of the buffer was added, filtered through a 40 μm nylon mesh, and stained by adding 6.5 μL of propidium iodide solution and 3.5 μl RNAse and analyzed after 15 min. The analyses were performed on a FACSCalibur (BD Biosciences, San José, USA) cytometer at Universidade Federal de Ouro Preto, equipped with a laser source (488 nm) and the histograms were obtained by the BD Cell Quest software. For each sample, at least 10,000 nuclei were analyzed regarding their relative fluorescence intensity. Three independent replicates (three individuals per colony) were conducted and histograms with a coefficient of variation above 5% were rejected. Histograms were analyzed using the Flowing 2.5.1 software (http://www.flowingsoftware.com). The genome size of each *S.saevissima* was calculated using the 1C-value (0.18 pg) of *Drosophilamelanogaster* and the values were obtained according to the equation by Doležel and Bartos (2005) and converted to megabase pairs (1 pg = 978 Mbp).

## ﻿Results

**Figure 2.**
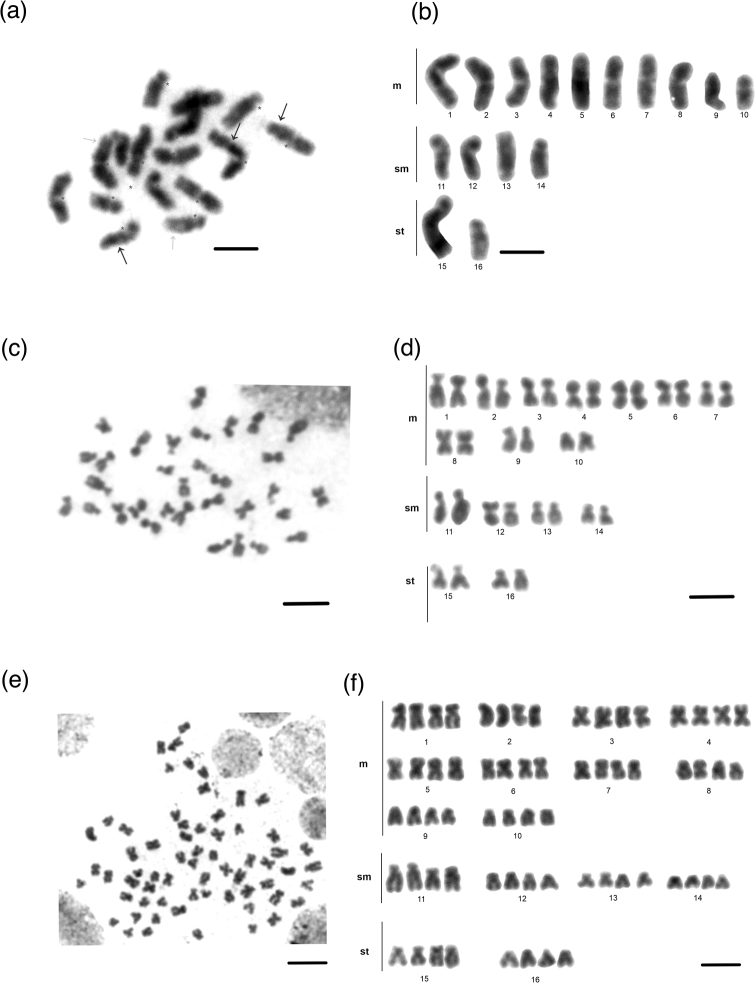
Chromosomes of *Solenopsissaevissima***a** metaphase **b** haploid karyotype; n = 16 **c** metaphase **d** diploid karyotype, 2n = 32 **e** metaphase; and **f** tetraploid karyotype, 4n = 64. Asterisks, grey and black arrows indicate centromeres as well as smaller and larger heterochromatic segments respectively. Scale bars: 10 µm.

**Figure 3.**
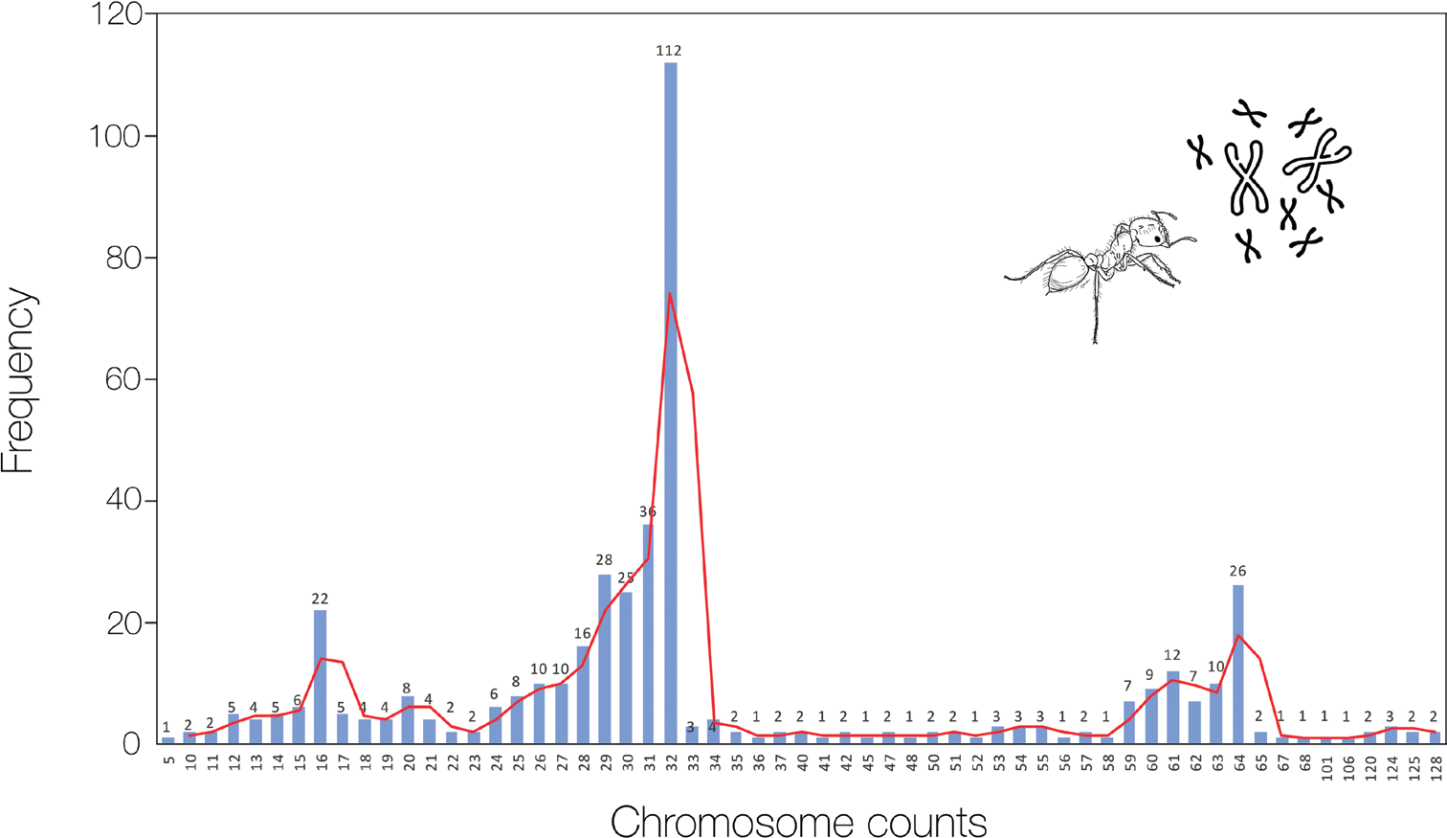
Chromosome count frequency of *Solenopsissaevissima* throughout all 452 metaphases. The highest frequency was observed in the modal haploid (n = 16) and diploid (2n = 32) karyotypes together with the less frequent 4n = 64. The red line represents the tendency curve. Variations are due to the technique employed to obtain mitotic chromosomes.

The chromosome counts for the *S.saevissima* individuals analyzed here were n = 16 (22 metaphases), 2n = 32 (122 metaphases), 4n = 64 (26 metaphases), and 8n = 128 (a single metaphase) (Fig. 2) considering all colonies. Our observations confirm that we can commonly find polyploid cells in the brain ganglion of immatures of *S.saevissima*. All counts (n = 452) are summarized in Fig. 3, showing the distribution of metaphases around the modal chromosome numbers 16, 32, and 64. The karyomorphometric data from haploid and diploid karyotypes are given in Suppl. material 1: tables S1, S2. Accurate karyomorphometric analysis from the polyploid metaphases was unlikely, but a particular 4n metaphase was evaluated and the resulting measurements are given in Suppl. material 1: table S3. The karyotypic formulas found were n = 10m + 4sm + 2st, 2n = 20m + 8sm + 4st, and 4n = 40m + 16sm + 8st. The two largest metacentric and submetacentric chromosome pairs showed secondary constrictions. Polyploid cells were observed in all colonies at the similar frequency (Suppl. material 1: table S4).

**Figure 4.**
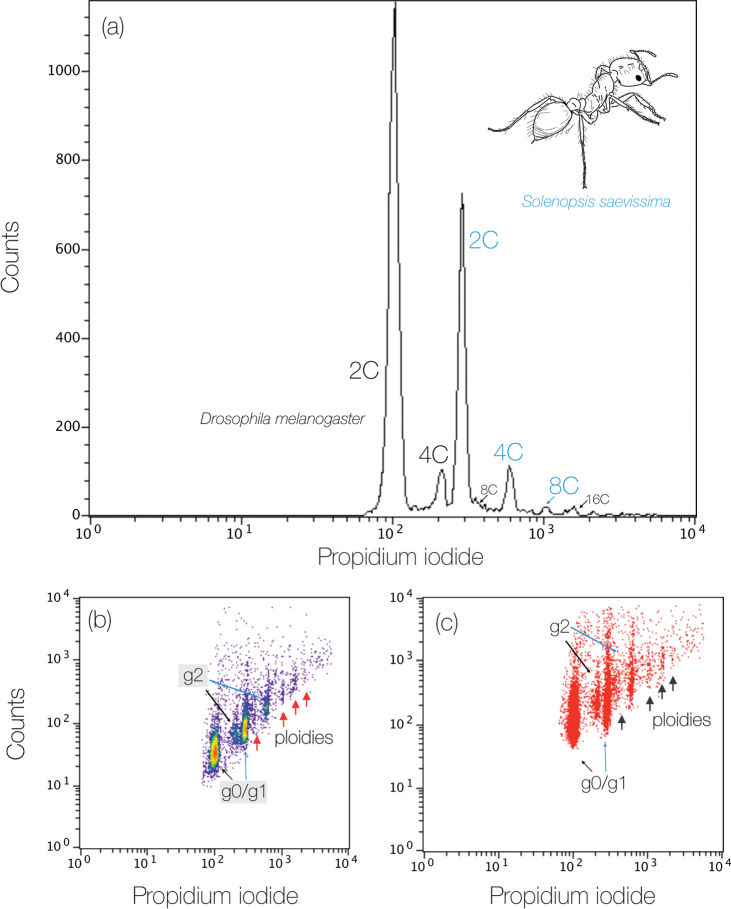
Genome size of *Solenopsissaevissima* showing ploidy variations estimated by flow cytometry **a** histogram highlighting the peaks from 2C to 8C (blue lettering refers to *S.saevissima* and black lettering refers to the internal standard) **b** density plot **c** dot plot containing many events, within which the nuclei occupy a well-defined series of regions, equally spaced in terms of fluorescence and corresponding to 2C, 4C, 8C, and 16C nuclei. Colors in the density plot indicate nuclei population density, with red as the highest and blue as the lowest.

The nuclei isolated from the brain tissue were properly recovered given the histograms showing peaks from cells at different stages of the cell cycle: the higher peak G0/G1 (unreplicated DNA in the nuclei – 2C) and lower peak G2 (replicated DNA – 4C). Additional peaks were observed after the common G0/G1 and G2 peaks for which the nuclei occupy a well-defined series of regions, equally spaced in terms of fluorescence and corresponding to 8C and 16C nuclei (see Fig. 4). The population of nuclei declines from 2C to 16C, representing the other ploidies observed both for the internal standard *D.melanogaster* as well for *S.saevissima*, indicating endoreduplication or polyploid cells as expected (Fig. 4). The genome size of *S.saevissima* was 0.51 ± 0.015 pg or 498.78 Mbp.

## ﻿Discussion

Here we observed a chromosome number variation in *S.saevissima* from n = 16 to 8n = 128 chromosomes. These counts agree with previous descriptions (Murakami et al. 2021). The typical chromosome number recovered from other *Solenopsis* species, such as *Solenopsisgeminata* (Fabricius, 1804), *Solenopsisrichteri* Forel, 1909, and *Solenopsisinvicta* is n = 16 (Cardoso et al. 2018a), which suggests that the chromosome number of n = 16 was the regular count of the haploid karyotype of *S.saevissima*. The other description from Uruguay also reported the same chromosome number (Goñi et al. 1983). The genome size estimates agree with previous data (Moura et al. 2021), and the 2C, 4C, and 8C values were clearly recovered by our flow cytometry analysis. Here we demonstrated that ploidy of cells varies among individuals within the colonies. Polyploid cells have been reported in other ants (see the reviews by Crozier 1975; Imai et al. 1977; and Lorite and Palomeque 2010), but not often. For example, although regularly studied from a cytogenetic point of view, polyploidy was not evidenced among fungus-farming ants (Cardoso and Cristiano 2021) or recovered by flow-cytometry studies (Moura et al. 2021), suggesting that polyploid cells may be restricted to some ant linages and not widespread within Formicidae. However, the lack of polyploid records may be due to the small coverage of cytogenetic studies and the high diversity of ant species. In her doctoral thesis, Silva (2016) used flow cytometry to demonstrate that there is a reversion of the presence of polyploid cells throughout the developmental stages of *S.saevissima* from larvae to pupae and adult workers, suggesting that polyploid cells occur only in the immature phase. This is expected since ants are holometabolous insects that do not change after metamorphosis. Thus, we hypothesized that the presence of polyploid cells in the immature and mutable stages may contribute to the colony’s fitness advantage.

The polyploid cells observed in the brood phase of *S.saevissima* may promote some benefit resulting in the developmental rate of the immature workers, which in turn will result in the number of workers. This could be analogous to the way polyploid cells occur in the salivary tissue of dipterans (Rodman 1967; Wells and Andrew 2015), who depend on salivary secretions to feed. The colony can grow faster and exploit available resources by reaching maturity for reproduction. *Solenopsissaevissima* is a recruiting species, and workers signal and convene other workers at resources (Yong-Yue et al. 2012), which can be exploited faster and invested into growing the colony and ultimately sexuals for reproduction (see Peeters and Molet 2010 for ant colony life cycle details). The faster a colony grows and exploits the environment to produce sexuals that will establish new colonies, the higher the fitness. Here, ploidy is indicated as a potential cytogenetic feature that allows *S.saevissima* to spread and colonize new areas, but such an idea requires experimental testing in future field studies.

Considering the stage where polyploid cells were found, it apparently results from endomitosis, which consists of normal G1 and G2 phases, but with incomplete mitosis. This means that the cytokinesis step does not occur at the end of the cell cycle, the chromosomes accumulate, thus generating polyploid cells (Lee et al. 2008). Indeed, studies in animal and even plant developmental systems have revealed conserved mechanisms that control the generation of polyploidy, and a reasonable expectation is that polyploid cells, through endoreplication, may provide key biological functions during developmental stages (Fox and Duronio 2013).

A recent study on *Solenopsis* by Murakami et al. (2021) compared species in native and invaded areas. Their results showed differences in chromosomal morphology between the analyzed populations, mainly in ploidy, suggesting a possible generalized hybridization between ants native to South and North America. Evidence of hybridization in this genus has already been reported by Taber and Cokendolpher (1988) and Ross and Shoemaker (2005). The former suggests that species in the US can hybridize with *S.invicta*, *S.geminata*, and *S.molesta* (Say, 1836). Hybridization in genetically close species can generate disarrangements in the cytoplasm, duplicating the genome and consequently resulting in polyploidy (Fujiwara et al. 1997).

Based on cytogenetic evidence, Murakami et al. (2021) suggested that invasive *Solenopsis* species, when settling in new environments, hybridize with closely related, or even genetically distant species. This process resulted in various chromosome numbers. Such a mechanism may promote an increase in the genetic diversity of the population and the acquisition of adaptive genes that will better acclimate species to the invaded environment (Chen 2010).

Our study complements the importance of understanding the chromosomal biology of ants. This approach can also help understand species’ life histories and contributes to the analysis of invasive species. Here, we found cytogenetic evidence that may reflect the species’ biology. *Solenopsis* ants are aggressive competitors, opportunistic scavengers nesting in open areas in urban and natural preserved environments (Lofgren et al. 1975) and are well-adapted to anthropized areas.

The external morphologies of *S.saevissima* and its congeners do not provide suitable traits to recognize potential cryptic species (Fox et al. 2012). Thus, karyotyping determines the number and morphology of chromosomes, proving to be a good tool for understanding genetic barriers within inconspicuous groups (Cristiano et al. 2017; Cardoso et al. 2018b). In the present study, cytogenetic analysis of *Solenopsissaevissima* yielded the same chromosome number, which was observed previously. Further, it appears that a chromosome number of n = 16 is a common karyotype feature of *Solenopsis* spp.

## ﻿Data availability statement

All relevant data are within the manuscript and its Supporting Information files (Suppl. material 1). All other information can be requested from the corresponding authors.

## Supplementary materials

Supplementary material 1 Results from the karyomorphometrical analyses of *Solenopsissaevissima* and Chromosome counts frequency by individual and colony of *Solenopsissaevissima* Data type tables (PDF file) Explanation note table S1: Results from the karyomorphometrical analyses of *Solenopsissaevissima* n = 16 chromosomes. TL: total length; L: long arm length; S: short arm length; RL: relative length; *r*: arm ratio (= L/S); m: metacentric; sm: submetacentric; st: subtelocentric; table S2: Results from the karyomorphometrical analyses of *Solenopsissaevissima* 2n = 32 chromosomes. TL: total length; L: long arm length; S: short arm length; RL: relative length; *r*: arm ratio (= L/S); m: metacentric; sm: submetacentric; st: subtelocentric; table S3: Results from the karyomorphometrical analyses of *Solenopsissaevissima* 4n = 64 chromosomes. TL: total length; L: long arm length; S: short arm length; RL: relative length; *r*: arm ratio (= L/S); m: metacentric; sm: submetacentric; st: subtelocentric; table S4; Chromosome counts frequency by individual and colony of *Solenopsissaevissima*. https://binary.pensoft.net/file/836444This dataset is made available under the Open Database License (http://opendatacommons.org/licenses/odbl/1.0/). The Open Database License (ODbL) is a license agreement intended to allow users to freely share, modify, and use this Dataset while maintaining this same freedom for others, provided that the original source and author(s) are credited.Ananda Ribeiro Macedo de Andrade, Danon Clemes Cardoso, Maykon Passos Cristiano
