# Supplementary material for: ﻿Assessing ploidy levels and karyotype structure of the fire ant Solenopsissaevissima Smith, 1855 (Hymenoptera, Formicidae, Myrmicinae)
Source: Comp Cytogenet. 2023 Apr 11;17:59–73. doi: 10.3897/compcytogen.17.100945 (PMC10696605; doi:10.3897/compcytogen.17.100945)
Supplement: Supplementary material 1 — Results from the karyomorphometrical analyses of Solenopsissaevissima and Chromosome counts frequency by individual and colony of Solenopsissaevissima [file comparative_cytogenetics-17--059_article-100945__-s001.pdf]

Supplementary tables

Table S1 - Results from the karyomorphometrical analyses of *Solenopsis saevissima* n = 16 chromosomes. TL: total length; L: long arm length; S: short arm length; RL: relative length; *r*: arm ratio (= L/S); m: metacentric; sm: submetacentric; st: subtelocentric.

	**TL**	**L**	**S**	**RL**	***r***	**Morphology**
1	4.46±1.03	2.5±0.6	1.96±0.51	8.73±0.54	1.3±0.24	m
2	3.97±0.85	2.21±0.48	1.76±0.47	7.81±0.52	1.31±0.4	m
3	3.86±0.91	2.1±0.46	1.76±0.47	7.54±0.421	1.22±0.16	m
4	3.54±0.81	2.03±0.43	1.5±0.53	6.95±0.45	1.51±0.72	m
5	3.46±0.94	1.94±0.53	1.52±0.43	6.73±0.7	1.28±0.14	m
6	3.43±0.8	1.85±0.49	1.58±0.34	6.73±0.67	1.16±0.16	m
7	3.1±0.82	1.75±0.5	1.34±0.38	6.04±0.69	1.34±0.3	m
8	3.04±0.71	1.69±0.38	1.35±0.45	5.96±0.6	1.4±0.75	m
9	2.71±0.96	1.47±0.44	1.23±0.53	5.23±1.08	1.24±0.2	m
10	2.41±0.75	1.33±0.41	1.08±0.38	4.73±1.07	1.26±0.31	m
11	3.46±0.76	2.42±0.61	1.04±0.22	6.84±1.09	2.35±0.53	sm
12	3.15±0.72	2.12±0.52	1.02±0.22	6.2±0.73	2.07±0.29	sm
13	2.6±0.65	1.79±0.47	0.8±0.22	5.08±0.49	2.33±0.64	sm
14	2.3±0.56	1.55±0.41	0.75±0.18	4.56±0.83	2.06±0.36	sm
15	3.02±1.11	2.41±0.96	0.6±0.18	5.84±1.47	3.98±1.04	st
16	2.53±0.63	1.91±0.57	0.61±0.16	4.95±0.63	3.25±1.07	st
**KL**	51.1					

Table S2 - Results from the karyomorphometrical analyses of *Solenopsis saevissima* 2n = 32 chromosomes. TL: total length; L: long arm length; S: short arm length; RL: relative length; *r*: arm ratio (= L/S); m: metacentric; sm: submetacentric; st: subtelocentric.

	**TL**	**L**	**S**	**RL**	***r***	**Morphology**
1	2.86±0.71	1.57±0.40	1.28±0.34	4.14±0.29	1.23±0.19	m
1	2.77±0.62	1.52±0.36	1.24±0.29	4.03±0.21	1.23±0.17	m
2	2.56±0.64	1.37±0.32	1.19±0.33	3.71±0.22	1.16±0.15	m
2	2.63±0.69	1.45±0.40	1.18±0.32	3.82±0.29	1.23±0.18	m
3	2.43±0.70	1.35±0.40	1.07±0.32	3.51±0.38	1.27±0.18	m
3	2.60±0.80	1.42±0.40	1.16±0.42	3.72±0.44	1.25±0.15	m
4	2.33±0.67	1.30±0.41	1.02±0.28	3.37±0.41	1.27±0.21	m
4	2.23±0.56	1.19±0.26	1.04±0.3	3.24±0.32	1.16±0.11	m
5	2.24±0.66	1.21±0.42	1.02±0.26	3.23±0.35	1.17±0.20	m
5	2.18±0.62	1.16±0.34	1.02±0.29	3.15±0.36	1.14±0.10	m
6	2.13±0.53	1.13±0.26	0.99±0.28	3.1±0.39	1.16±0.20	m
6	2.04±0.60	1.18±0.36	0.86±0.24	2.95±0.31	1.37±0.18	m
7	2.11±0.60	1.17±0.34	0.93±0.27	3.03±0.28	1.25±0.13	m
7	2.14±0.62	1.17±0.35	0.97±0.27	3.08±0.33	1.19±0.14	m
8	1.88±0.53	1.07±0.36	0.8±0.18	2.73±0.38	1.34±0.24	m
8	1.95±0.56	1.07±0.36	0.88±0.23	2.82±0.3	1.19±0.23	m
9	2.04±0.62	1.12±0.39	0.91±0.24	2.95±0.47	1.23±0.20	m
9	1.87±0.35	1.05±0.21	0.82±0.15	2.77±0.42	1.28±0.19	m

	TL	L	S	RL	*r*	Morphology
10	1.51±0.44	0.84±0.21	0.67±0.23	2.18±0.29	1.28±0.16	m
10	1.50±0.48	0.85±0.26	0.65±0.23	2.17±0.31	1.35±0.21	m
11	2.63±0.67	1.80±0.49	0.82±0.21	3.82±0.41	2.19±0.37	sm
11	2.55±0.66	1.75±0.48	0.80±0.21	3.70±0.44	2.21±0.35	sm
12	2.10±0.52	1.41±0.34	0.68±0.20	3.06±0.50	2.14±0.4	sm
12	2.06±0.53	1.39±0.37	0.66±0.19	2.99±0.43	2.14±0.39	sm
13	1.87±0.47	1.28±0.34	0.58±0.12	2.71±0.36	2.2±0.19	sm
13	1.89±0.51	1.28±0.36	0.60±0.16	2.74±0.49	2.14±0.25	sm
14	1.84±0.48	1.25±0.32	0.58±0.18	2.69±0.61	2.17±0.27	sm
14	1.76±0.41	1.20±0.29	0.56±0.12	2.57±0.4	2.14±0.14	sm
15	2.53±0.65	1.97±0.55	0.55±0.12	3.66±0.39	3.54±0.5	st
15	2.56±0.79	2.02±0.64	0.54±0.17	3.68±0.39	3.76±0.57	st
16	1.77±0.82	1.36±0.62	0.40±0.19	2.47±0.53	3.42±0.37	st
16	1.79±0.76	1.37±0.59	0.41±0.18	2.51±0.42	3.4±0.92	st
**KL**	69.4742481					

Table S3 - Results from the karyomorphometrical analyses of *Solenopsis saevissima* 4n = 64 chromosomes. TL: total length; L: long arm length; S: short arm length; RL: relative length; *r*: arm ratio (= L/S); m: metacentric; sm: submetacentric; st: subtelocentric.

	TL	L	S	RL	r	Morphology
1	2.15	1.29	0.86	1.00	1.50	m
1	2.23	1.37	0.87	1.03	1.58	m
1	2.23	1.33	0.90	1.03	1.48	m
1	2.21	1.39	0.82	1.02	1.69	m
2	2.15	1.15	1.01	1.00	1.14	m
2	1.73	0.95	0.78	0.80	1.22	m
2	1.87	0.94	0.93	0.86	1.01	m
2	1.79	1.11	0.68	0.83	1.63	m
3	1.98	1.09	0.89	0.92	1.23	m
3	2.06	1.08	0.98	0.95	1.11	m
3	1.66	0.85	0.81	0.77	1.04	m
3	1.83	0.98	0.85	0.85	1.16	m
4	1.85	1.00	0.84	0.85	1.19	m
4	1.89	1.04	0.85	0.88	1.22	m
4	1.89	1.00	0.89	0.88	1.13	m
4	2.13	1.14	0.99	0.99	1.15	m
5	1.94	1.01	0.93	0.90	1.08	m
5	2.00	1.10	0.90	0.92	1.23	m
5	1.90	1.05	0.85	0.88	1.23	m
5	1.94	0.98	0.96	0.90	1.02	m
6	1.76	0.99	0.77	0.82	1.28	m
6	1.73	1.04	0.70	0.80	1.48	m
6	1.90	0.99	0.91	0.88	1.09	m
6	1.93	0.99	0.94	0.89	1.05	m
7	1.85	1.03	0.82	0.86	1.26	m
7	1.68	0.93	0.75	0.78	1.24	m

	TL	L	S	RL	r	Morphology
7	1.56	0.81	0.75	0.72	1.09	m
7	1.60	0.87	0.73	0.74	1.20	m
8	1.62	0.97	0.65	0.75	1.48	m
8	1.62	0.88	0.74	0.75	1.19	m
8	1.62	0.87	0.75	0.75	1.16	m
8	1.43	0.89	0.54	0.66	1.63	m
9	1.43	0.89	0.54	0.66	1.64	m
9	1.65	0.83	0.82	0.76	1.00	m
9	1.54	0.93	0.61	0.71	1.52	m
9	1.35	0.77	0.58	0.62	1.34	m
10	1.32	0.74	0.59	0.61	1.25	m
10	1.35	0.82	0.52	0.62	1.57	m
10	1.35	0.78	0.57	0.63	1.36	m
10	1.20	0.65	0.55	0.56	1.17	m
11	2.49	1.82	0.67	1.15	2.74	sm
11	2.70	1.94	0.76	1.25	2.53	sm
11	2.08	1.47	0.61	0.96	2.40	sm
11	2.02	1.41	0.61	0.94	2.29	sm
12	1.41	1.01	0.39	0.65	2.58	sm
12	1.46	0.99	0.47	0.68	2.11	sm
12	1.47	0.97	0.50	0.68	1.96	sm
12	1.30	0.84	0.47	0.60	1.80	sm
13	1.40	0.90	0.50	0.65	1.80	sm
13	1.43	0.93	0.50	0.66	1.85	sm
13	1.31	0.84	0.47	0.61	1.81	sm
13	1.29	0.83	0.46	0.59	1.79	sm
14	1.05	0.68	0.37	0.49	1.87	sm
14	0.97	0.71	0.26	0.45	2.67	sm
14	1.19	0.84	0.35	0.55	2.41	sm
14	1.06	0.76	0.30	0.49	2.54	sm
15	1.98	1.49	0.49	0.92	3.05	st
15	1.71	1.29	0.42	0.79	3.10	st
15	1.86	1.42	0.44	0.86	3.25	st
15	1.79	1.42	0.38	0.83	3.77	st
16	1.39	1.06	0.32	0.64	3.32	st
16	1.50	1.17	0.33	0.69	3.57	st
16	1.31	1.02	0.2293	0.61	3.52	st
16	1.32	1.01	0.31	0.61	3.23	st
**KL**	215.98					

Table S4 – Chromosome counts frequency by individual and colony of *Solenopsis saevissima*.

Colony	Counting date	Slide/Individual	n chromosomes	frequency	ploidy
N1	22/10/2020	L3	13	1	
			19	1	
			30	1	
N1	22/10/2020	L6	10	1	
			11	1	
			12	2	
			13	1	
			14	1	
			16	3	1n
			18	1	
			19	1	
			20	1	
			23	1	
			24	1	
			25	2	
			26	4	
			27	1	
			28	4	
			29	1	
			30	3	
			31*	4	
			32*	20	2n
			33	1	
			34	1	
			40	1	
			47	1	
			50*	2	
			51	1	
			53*	2	
			54	1	
			57	1	
			59	4	
			60*	3	
			61*	2	
			62	1	
			63	3	
			64	2	4n
			124	1	
			128	1	8n
N1	22/10/2020	L7	16	1	1n
			17	1	
			21	1	

Colony	Counting date	Slide/Individual	n chromosomes	frequency	ploidy
			23	1	
			31	1	
			32	3	2n
			29*	1	
N1	26/10/2020	L1	12	1	
			20	1	
			30	1	
			31	1	
			32	4	2n
N1	26/10/2020	L2	20	1	
			21	1	
			22	1	
			24	1	
			25	1	
			28	1	
			29	6	
			30	1	
			32	4	2n
			61	2	
N1	26/10/2020	L3	20	1	
			21	1	
			27	1	
			28	1	
			29	1	
			30	1	
			32	11	2n
N1	26/10/2020	L4	5	1	
			10	1	
			19	1	
			24	1	
			28	1	
			30	3	
			31	1	
			32	5	2n
			48	1	3n
			54	1	
			60	1	
			63	1	
			64	1	4n
			65	1	
N3	01/04/2021	L8	27	1	
			30	2	
			32	1	2n

Colony	Counting date	Slide/Individual	n chromosomes	frequency	ploidy
			64	1	4n
N4	17/11/2021	L3	30	2	
			31	1	
			32	4	2n
			34	1	
N4	17/11/2021	L5	12	1	
			16	2	1n
			19	1	
			20	1	
			27	1	
			28	1	
			29	3	
			31	2	
			32	2	2n
			60	1	
			64	3	4n
			120	1	
			128	1	8n
N4	17/11/2021	L6	28	2	
			29	7	
			30	2	
			31	8	
			32	19	2n
			33	1	
			34	1	
			35	1	
			41	1	
			42	1	
			45	1	
			47	1	
			51	1	
			53	1	
			55	3	
			59	1	
			60	2	
			61	4	
			62	2	
			63	2	
			64	6	4n
			101	1	
			106	1	
			120	1	
			124	1	

Colony	Counting date	Slide/Individual	n chromosomes	frequency	ploidy
			125	1	
			128	1	8n
N4	17/11/2021	L8	11	1	
			13	2	
			14	3	
			15	5	
			16	16	1n
			17	4	
			18	2	
			20	2	
			21	1	
			24	2	
			25	3	
			26	3	
			27	2	
			28	5	
			29	4	
			30	2	
			31	9	
			32	10	2n
			35	1	
			36	1	
			37	1	
			40	1	
			52	1	
			54	1	
			56	1	
			57	1	
			58	1	
			59	2	
			60	2	
			61	4	
			62	4	
			63	2	
			64	13	4n
			65	1	
			68	1	
			124	1	
			125	1	
N5	08/12/2021	L2	12	1	
			14	1	
			15	1	
			18	1	

Colony	Counting date	Slide/Individual	n chromosomes	frequency	ploidy
			20	2	
			22	1	
			24	1	
			26	2	
			27	2	
			28	1	
			29	3	
			30	5	
			31	7	
			32	17	2n
			34	1	
			42	1	
			63	1	
			64	1	4n
N5	10/12/21	L4	25	1	
			26	1	
			27	2	
			29	1	
			30	2	
			31	1	
			32	7	2n
			42	1	
			64	1	4n
N5	10/12/21	L5	25	1	
			32	4	2n
			33	1	
			67	1	
N5	10/12/21	L8	29	1	
			30	1	
			31	1	
			32	2	2n
			37	1	
